# Resource Security Impacts Men’s Female Breast Size Preferences

**DOI:** 10.1371/journal.pone.0057623

**Published:** 2013-03-06

**Authors:** Viren Swami, Martin J. Tovée

**Affiliations:** 1 Department of Psychology, University of Westminster, London, United Kingdom; 2 Department of Psychology, HELP University College, Kuala Lumpur, Malaysia; 3 Institute of Neuroscience, Newcastle University, Newcastle-upon-Tyne, United Kingdom; Colorado State Univeresity, United States of America

## Abstract

It has been suggested human female breast size may act as signal of fat reserves, which in turn indicates access to resources. Based on this perspective, two studies were conducted to test the hypothesis that men experiencing relative resource insecurity should perceive larger breast size as more physically attractive than men experiencing resource security. In Study 1, 266 men from three sites in Malaysia varying in relative socioeconomic status (high to low) rated a series of animated figures varying in breast size for physical attractiveness. Results showed that men from the low socioeconomic context rated larger breasts as more attractive than did men from the medium socioeconomic context, who in turn perceived larger breasts as attractive than men from a high socioeconomic context. Study 2 compared the breast size judgements of 66 hungry versus 58 satiated men within the same environmental context in Britain. Results showed that hungry men rated larger breasts as significantly more attractive than satiated men. Taken together, these studies provide evidence that resource security impacts upon men’s attractiveness ratings based on women’s breast size.

## Introduction

Given the human propensity to ‘judge books by their covers’ and the psychosocial impact of doing so [1], it is not surprising that scholars have attempted to delineate the physical features that lead to differential perceptions and treatment. In terms of women’s physical attractiveness, for example, a good deal of research has focused on the relative importance of traits such as body size and shape, leg length, hair colour and length, skin tone, and facial features [2]–[3]. By contrast, much less scholarly research has focused on women’s breasts, despite the sexual significance of breasts in most human societies [4]–[5]. Indeed, eye-tracking studies have indicated that, when judging the attractiveness of a woman, both men and women spend more time looking at the breasts and upper-body than any other bodily region [6]–[7].

Despite such evidence, the significance of prominent female breasts has proved difficult to explain from an evolutionary perspective, particularly as the human female is the only primate that has permanent, full-form breasts when not pregnant [8]. Theories that currently lack reliable evidence include the suggestion that the breast served functional roles such as milk storage for breast-feeding babies [9], comfort for nursing infants [10], and heat stress avoidance [11]. On the other hand, it is possible that biomechanical constraints as a result of sexually dimorphic fat deposition placed unique demands on human female morphology, which resulted in the selection of breasts [12]. Once enlarged, sexual selection may have enhanced the expression of permanently enlarged breasts [13], with breasts variously argued to act as a sign of nulliparity, age, sexual maturity, or fertility [14]–[17].

Based on this perspective, it has been proposed that men should find larger breasts more physically attractive, which appears consistent with the objectification and fetishisation of large breasts in post-industrial societies [18]–[19]. However, studies that have tested this hypothesis have returned mixed findings, with evidence of a preference for small [20], medium [21]–[24], and large breasts [25]–[27]. This inconsistency can be partly explained as a function of the presentation format of stimuli (e.g., frontal versus side-view) [28] and the poor ecological validity of line-drawn figures used in earlier studies [29]. When photographic and computer-generated stimuli are used instead, it appears that men in post-industrial societies show a preference for medium-to-large breasts [7], [28], [30].

An additional problem is that previous studies have not fully accounted for both within- and cross-cultural differences in men’s breast size judgements. In the first instance, it has been reported that larger breasts are preferred by men pursuing low-commitment, transient sexual relationships [28] and holding stronger sexist attitudes [30]. Additionally, cross-cultural differences in breast size preferences have been reported [31]–[32], with men in environments experiencing relative resource insecurity generally showing a stronger preference for larger breasts than their counterparts in contexts of relative resource security [33]. Based on these findings, it might be possible to conclude that one function of breasts is to act as an honest signal of fat reserves in non-lactating women [15], [34]–[35], which in turn indicates access to food or resources.

This perspective is consistent with the fact that the human breast is partly composed of adipose tissue, the distribution of which varies between women [36] but not between breasts within women [37]. Although the amount of adipose tissue varies relative to glandular tissue (e.g., during lactation) [38], human females are unique compared to other species, where the adipose tissue of the mammary gland is situated mainly in subcutaneous and abdominal regions [39]. By contrast, breast size in human female appears to be more strongly correlated with the amount of adipose tissue rather than mammary tissue [40]–[41]. In addition, environmental factors have been implicated in female breast size, particularly energy intake in early life [42]–[43], and the genetic contribution to breast size is largely unique to this phenotype and not shared with body mass index [44]. Combined with their prominent display and pendulous morphology, it is possible that the female breast functions, partly at least, as an indicator of adipose tissue storage.

In this view, men in situations marked by resource insecurity or uncertainty will be expected to idealise larger female breasts, as large size would be an honest signal of access to resources, so long as the amount of fat is not so great as to detract from an appearance of high reproductive value or be maladaptive. To date, however, there have been no systematic tests of this hypothesis and existing evidence comes purely from data gathered in naturalistic settings [32]–[33]. To overcome this dearth in the literature, we conducted a systematic investigation of the hypothesis that relative resource security impacts upon men’s perceptions of women’s attractiveness based on breast size. In Study 1, we examined differences in perceptions of attractiveness based on breast size among men from different socioeconomic contexts, whereas in Study 2 we investigated the impact of hunger on breast size judgements among men from the same environmental context.

## Study 1

Study 1 examined whether there are systematic differences in attractiveness judgements based on breast size among men from the same national, but different socioeconomic, contexts. Certainly, the available evidence suggests that there are reliable differences in body size judgements as a function of socioeconomic status, with men from low socioeconomic sites showing a stronger preference for heavier women than men from high socioeconomic sites [45]–[50]. In addition, men from the former sites also appear to rate overweight and obese women more positively than do men from high socioeconomic contexts [45]–[49]. Similar findings have been reported when women from different socioeconomic contexts are asked to rate the attractiveness of men varying in body size [51], suggesting that the effect is gender-invariant.

The available evidence also points to similar differences in terms of breast size judgements. In one study, it was reported that men from relatively impoverished and isolated sites in Papua New Guinea preferred larger breast size to a greater extent than men from Samoa and New Zealand [33]. However, it is possible that this finding is confounded by intra-national and inter-national differences that impact on breast size judgements, such as attitudes toward women [30]. A more conclusive test of whether breast size judgements vary as a function of socioeconomic contexts would be aided by sampling men from the same national context, but from different socioeconomic contexts, as has been done with body size judgements [45]–[49]. As such, in Study 1, we examined breast size judgements of Malaysian men from different socioeconomic sites.

### Method

#### Ethical statement

The ethics committee at the Department of Psychology, University of Westminster, specifically approved this study. All participants provided written informed consent.

### Participants

#### Study site

The study site for this study was the state of Sabah, Malaysia, on the island of Borneo. Compared to other Malaysian states, Sabah remains one of the least developed (GDP per capita about US$2,400), with average annual incomes being the lowest in the country [52]. However, development in the state is highly uneven, resulting in large intra-state disparities in socioeconomic status. The state capital, Kota Kinabalu, is a large conurbation that received city status in 2000 and is inhabited by an ethnically mixed population of just under half a million. The city serves as the commercial and industrial hub of the state and has also emerged as the main tourist gateway to the island of Borneo. By contrast, the interior of the state remains largely impoverished, with small townships and villages where agriculture and tourism remain the primary source of income.

In the present study, we recruited participants from Kota Kinabalu (high socioeconomic status), the township of Ranau (medium socioeconomic status), and three villages in the West Coast Administrative Division of Sabah (low socioeconomic status). Ranau is a small township about 100 km east of Kota Kinabalu, with a population of just over 10,000 and where the main source of income is vegetable farming. The three village sites were located at least 50 km from Ranau and were relatively isolated, with permanent mains water and electricity supplies but limited access to mass media. Previous studies have made use of a similar socioeconomic gradient in the state of Sabah [45], [51], [53]–[55]. Although there are unlikely to be major differences in attractiveness judgements as a function of ethnic group in this context [44], [51], we nevertheless only recruited Kadazan participants, who are the majority ethnic group in Sabah.

#### Participants

Participants from Kota Kinabalu were 102 men employed in various tertiary industries (e.g., tourism-related and service sectors), with a mean age of 42.01 years (*SD* = 13.08) and mean body mass index (BMI) of 21.92 kg/m^2^ (*SD* = 4.20). The majority of participants were Roman Catholics (89.2%; Protestant = 9.8%; other = 1.0%) and had completed secondary education (78.4%; undergraduate degree = 19.6%; postgraduate degree = 2.0%). Participants from the township of Ranau were 87 vegetable farmers (age *M* = 42.82, *SD* = 11.72; BMI *M* = 22.02, *SD* = 4.54), the majority of whom were Roman Catholics (82.8%; Protestant = 13.8%; other = 3.3%). The vast majority of participants in this group had completed secondary education (92.0%; primary education = 4.6%; undergraduate degree = 3.4%). Finally, participants from the three target villages were 77 subsistence farmers with a mean age of 40.81 years (*SD* = 13.21) and a mean BMI of 22.77 kg/m^2^ (*SD* = 3.68). Participants in this final group were mainly Roman Catholics (85.7%; Protestant = 10.4%; other = 3.9%) who had completed secondary education (79.2%; primary = 20.8%).

### Materials

#### Breast size preferences

**Figure 1:**
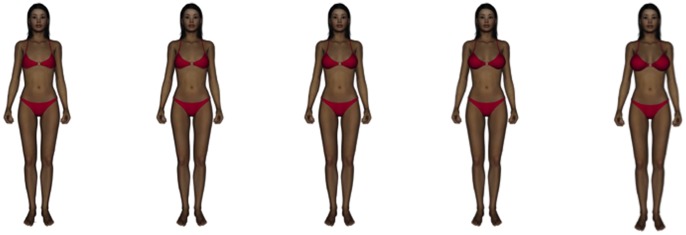
Stimuli used in Study 1. *Note*. During presentation, the stimuli rotated in 360°.

To assess attractiveness perceptions based on breast size, we followed previous work [30] in creating three-dimensional (3D) animations of female figures that were allowed to rotate through 360° relative to the viewer. Doing so allowed us to avoid known limitations of presenting stimuli from a single viewing angle [28] and also enhances ecological validity of the presentation method. The stimuli were created using Daz Studio 3.1 (www.daz3d.com), which enables users to create photo-realistic 3D models. For the present study, we used the Victoria 4.2 female model modified with the RM_Mylin for V4 face and body shape, with the Marikit for V4.2 skin texture, the Victoria 4 bikini, and Glamour Hair V4 (with the black hair texture option). We selected these characteristics as they most closely matched the ethnic group being studied, a procedure that has been used previously [33]. Consistent with previous work [30], breast size was set at five levels, namely −100, −50, 0, 50, and 100, using the breast size slider on Body morphs++ add-on package. This reflects an incremental change in cup size (i.e., the measurement around a woman’s torso over the fullest part of the breasts) without altering lower torso circumference. Each figure was rotated through 360° in 5° steps using the aniMate2 package, rendered in 24-bit colour and in 685×895 pixel resolution, and exported as 30-frames-per-second audio video interleaves. During testing, the stimuli were presented concurrently in ascending order on 13-inch laptop computers (see Figure 1). Participants in all sites were asked to rate the figure they found most physically attractive (1 = *Very small breast size*, 2 = *Small breast size*, 3 = *Medium breast size*, 4 = *Large breast size*, 5 = *Very large breast size*).

#### Financial security

Previous research in Malaysia has suggested that rural participants may not share the same understanding of poverty and income as their urban counterparts [56]. Combined with the fact that many individuals in rural settings do not receive a steady monthly income, it has been suggested that measures of actual income may not be a reliable measure of socioeconomic status in this context [54]. Following previous work [54], therefore, we asked participants to self-report their financial security compared to other Malaysians of their own age and gender (1 = *Less secure*, 2 = *Same*, 3 = *More secure*). **Body mass index**. Rural participants may not be able to accurately self-report BMI [54]. For this reason, we directly measured all participants’ body mass (kg) and height (cm) to the nearest 0.5 kg and 0.5 cm, respectively, using a standard tape measure and weighing scale. All participants were measured without shoes and in loose clothing. BMI for each participant was computed as kg/m^2^.

#### Demographics

All participants were asked to provide their age, religion, and highest educational qualification. Some rural participants were not able to precisely report their age and, in these cases, they were asked to estimate their age as accurately as they could.

### Procedure

Following established procedure [55], recruitment of participants began in rural villages. Permission was obtained from village heads to conduct a study ostensibly on health and appearance, and participants who agreed to take part in the study and who met eligibility criteria were given further information (survey information and participant rights) by a male researcher. Once participants provided informed consent, they were asked to view the breast size stimuli in a quiet and private location and make their ratings on a paper-and-pencil survey. They then completed the additional measures described above, before the same researcher directly obtained participants’ height and weight. Once data collection in rural sites was complete, age-matched samples of township and city participants were recruited from Ranau and Kota Kinabalu, respectively. The survey methods in both these sites were identical to that established in the rural sites. All participants completed the survey individually, took part on a voluntary basis, and were not remunerated for participation. All participants were verbally debriefed once testing was completed.

### Results and Discussion

Preliminary analyses using univariate analyses of variance (ANOVAs) showed that there were no significant between-group differences in participant age, *F*(2, 263) = 0.52, *p* = .597, η_p_^2^<.01, and BMI, *F*(2, 263) = 1.01, *p* = .365, η_p_^2^<.01. On the other hand, there was a significant between-group difference in the distribution of educational qualifications, χ^2^(6) = 54.66, *p*<.001, Φ = .45, with participants in Kota Kinabalu being more likely to hold higher qualifications than participants in the other sites. There was also a significant between-group difference in self-reported financial security, *F*(2, 263) = 29.14, *p*<.001, η_p_^2^ = .18. Tests of simple effects showed that participants in Kota Kinabalu reported significantly higher financial security than participants in Ranau, *t*(187) = 4.63, *p*<.001, *d* = 0.68, and rural villages, *t*(177) = 6.38, *p*<.001, *d* = 0.96. In addition, participants from Ranau reported being significantly more financially secure than their rural counterparts, *t*(162) = 3.73, *p*<.001, *d* = 0.59. The direction and strength of these differences are in accord with previous research [54].

Frequencies of ratings as a function of breast size and research site are reported in Table 1 along with skewness statistics. As can be seen, the figure with medium breast size was selected most frequently in Ranau and Kota Kinabalu, whereas the figure with large breast size was selected most frequently by rural participants. Furthermore, the skew toward larger breast size was more pronounced among rural participants than it was among participants in Ranau or Kota Kinabalu (see Table 1). A univariate ANOVA showed that there were significant between-group differences in the breast size rated as the most physically attractive, *F*(2, 263) = 11.31, *p*<.001, η_p_^2^ = .08 (descriptive statistics reported in Table 1). Tests of simple effects showed that rural participants rated a significantly larger breast size as more attractive than did participants in Ranau, *t*(162) = 2.44, *p* = .016, *d* = 0.38, and Kota Kinabalu, *t*(177) = 4.74, *p*<.001, *d* = 0.71. In addition, participants in Ranau rated a significantly larger breast size as more attractive than participants in Kota Kinabalu, *t*(187) = 2.32, *p* = 0.22, *d* = 0.34. We also examined the correlation between breast size preferences and relative financial security for the total sample in Study 1. Results indicated that lower financial security was associated with a preference for larger breast size, *r* = –.15, *p* = .014.

**Table 1:** Frequency of breast size rated as most physically attractive by research site, as well as skewness statistics.

		Site		
		Rural villages (*n* = 77)	Ranau (*n* = 87)	Kota Kinabalu (*n* = 102)
Breast size (%)	Very small	2.6	4.6	6.9
	Small	6.5	6.9	14.7
	Medium	14.3	34.5	42.2
	Large	44.2	33.3	21.6
	Very large	32.5	20.7	14.7
Shapiro-Wilk statistic		.83*	.89*	.90*
Skewness		−1.05	−0.49	−0.09
Kurtosis		.94	.01	−.39
Mean		3.97	3.58	3.23
Standard deviation		0.99	1.04	1.09

Note. *p<.001.

The results of Study 1 indicate that there are significant differences in judgements of women’s attractiveness based on breast size as a function of men’s relative socioeconomic status. More specifically, the present results indicate that men in relatively low socioeconomic sites rate larger breast sizes as more physically attractive than do their counterparts in moderate socioeconomic sites, who in turn rate a larger breast size as more attractive than individuals in a high socioeconomic site. In broad terms, these results are consistent with previous studies showing that there is an inverse relationship between socioeconomic status and breast [33] and body size [41]–[47] judgements. These results provide preliminary evidence that breast size may act as an indicator of calorific storage and that men in environments characterised by relative resource insecurity perceive larger breast sizes as more attractive than their counterparts in higher socioeconomic contexts.

## Study 2

An important limitation of Study 1 is the possibility that inter-regional differences partially account for the significant differences we observed. For example, combining the findings that men who hold greater sexist attitudes show a preference for larger breast size [30] and that patriarchal pressure may be greater in rural areas [45], it is possible that there was a natural confound in our design that limits the conclusions that can be made. One way in which this limitation could be overcome would be to focus on participants from the same environment but who differ along a dimension that acts as a proxy for resource security. One such dimension that has been proposed in the literature is proprioceptive hunger, with studies indicating that hungry men rate a significantly heavier female body size as attractive [57]–[59] and also positively idealise overweight and obese women compared to satiated men [59].

These findings have been explained as a function of environmental security [60]–[61]: when socioeconomic or individual conditions are insecure or threatening, individuals are hypothesised to idealise more mature physical characteristics, including heavier body size. It has been suggested that mature physical characteristics may signal ability to handle threatening environmental conditions or because they are honest indicators of traits (e.g., strength and independence) that are more desirable during periods of environmental insecurity [62]. Indeed, there is a good deal of evidence to support this perspective, including archival [60],[63] and empirical data [64]–[65] in humans, as well as non-human species [66]. To date, however, the impact of hunger on men’s breasts size preferences specifically has not been investigated.

If breast size does act as a reliable indicator of access to resources and calorific storage, then it should be expected that hungry men would show a preference for larger breast size than satiated men. More broadly, it is also possible that larger breasts size signals greater physical maturity, a trait that may be preferred under conditions of environmental insecurity. For example, it has been proposed that men may use breast size to gauge the age of a woman [17], with larger, non-sagging breasts signalling that a woman is mature but not old. Both of these perspectives lead to the prediction that hungry men will rate women with larger breasts as more attractive than satiated men, which we tested in Study 2.

### Method

#### Ethical statement

The ethics committee at the Department of Psychology, University of Westminster, specifically approved this study. All participants provided written informed consent.

### Participants and Procedure

The design of Study 2 followed closely the set-up for previous studies examining the impact of hunger on men’s body size judgements [57], [59], [67]. Male university students were asked to take part in the study as they entered or exited campus dining halls during dinner (approximately 6∶00 to 7∶00 pm) on a random selection of weekdays over the course of six weeks. Because participant ethnicity is known to affect breast size judgements [32], only British White men were invited to take part in this study. Participants were prevented from taking part in the study twice by two male researchers trained in psychological methods and by asking participants to provide a unique combination of their initials, date of birth, and mother’s maiden name (stored for the purposes of immediate cross-checking only and destroyed prior to any analyses). Potential participants were invited to take part on a study ostensibly on their health and eating habits (non-relevant, filler scales were included in the survey to mask the study’s purpose).

Upon being stopped, participants initially self-reported their hunger on a 7-point scale (1 = *Very hungry*, 2 = *Quite hungry*, 3 = *More hungry than full*, 4 = *More full than hungry*, 5 = *Quite full*, 6 = *Very full*, 7 = *Unsure*). In line with previous work [59], individuals who indicated a score of 1 or 2 were classified as hungry and those who reported a score of 5 or 6 were classified as satiated. Participants who gave a response other than these were asked to provide their age, height, and weight and were fully debriefed (*n* = 92; age *M* = 19.77, *SD* = 3.33; BMI *M = *21.57, *SD* = 3.68). The final sample consisted of 65 hungry participants (age *M* = 19.64, *SD* = 2.89; BMI *M* = 21.70, *SD* = 3.51) and 58 satiated participants (age *M* = 19.10, *SD* = 1.22; BMI *M* = 21.47, *SD* = 3.69). This final set of participants was tested individually in a quiet on-campus location and was debriefed once testing was completed.

### Materials

#### Breast size preferences

To assess breast size preferences, we used a previously developed set of 3D animations of female figures approximating Caucasian ethnic features [30]. The figures vary in five levels of breast size and rotated through 360°. As in Study 1, the stimuli were presented concurrently in ascending order on 13-inch laptop computers and participants were asked to rate the figure they found most physically attractive (1 = *Very small breast size*, 2 = *Small breast size*, 3 = *Medium breast size*, 4 = *Large breast size*, 5 = *Very large breast size*).

#### Appetite sensation

We obtained a subjective assessment of each individual’s appetite sensation using the Appetite Sensation Assessment [68]. This measure presents participants with 100 mm lines anchored at each end by words describing extremes of hunger, satiety, fullness, and prospective food consumption. Participants are asked to mark the line at the position on the scales corresponding to their feelings. Each item is scored by measuring the distance from the left end of the line to the mark. Finally, an overall score of satiety was computed as the mean of all four responses, with higher scores indicating greater hunger. This method of assessing appetite sensation has been shown to have good psychometric properties, including test-retest reliability and indices of validity [68]–[69].

#### Demographics

Participants self-reported their age, height, and weight. The latter two items were used to calculate participants’ BMI as kg/m^2^. Self-reported BMI has been shown to be very strongly correlated with actual BMI [70]–[71].

### Results and Discussion

Preliminary analyses using univariate ANOVAs showed that there were no significant differences between participants who were included and excluded from analyses in age, *F*(1, 213) = 1.21, *p* = .273, η_p_^2^<.01, and BMI, *F*(1, 213) = 1.59, *p* = .208, η_p_^2^<.01. These results suggest that our exclusion procedure did not unduly bias the retained sample. In addition, hungry participants were not significantly different from satiated participants in terms of age, *t*(121) = 1.33, *p* = .186, *d* = 0.24, and BMI, *t*(121) = 0.37, *p* = .714, *d* = 0.07. As expected, hungry participants reported significantly greater hunger on the Appetite Sensation Assessment than did satiated participants, *t*(121) = 10.55, *p*<.001, *d* = 1.92, indicating that our procedures were successful in distinguishing hungry and satiated individuals.

Examination of the breast size judgements indicated a greater skew toward larger breast size in the hungry group (Shapiro-Wilk statistic = .86, skewness = –.76, kurtosis = –.13) compared with the satiated group (Shapiro-Wilk statistic = .91, skewness = –.29, kurtosis = –.62). In the hungry group, 4.6% participants rated the very small breast size as the most attractive, 10.8% rated the small breast size, 18.5% the medium breast size, 36.9% large breast size, and 29.2% very large breast size. Equivalent frequencies for the satiated group were as follows: very small 8.6%, small 15.5%, medium 31.0%, large 29.3%, and very large 15.5%. An independent samples *t*-test indicated that the hungry men rated a significantly larger breast size as more physically attractive than did the satiated group (hungry *M* = 3.75, *SD* = 1.13; satiated *M* = 3.28, *SD* = 1.17), *t*(121) = 2.30, *p* = .023, *d* = 0.42.

The results of Study 2 indicate that hungry men rated a significantly larger female breast size as physically attractive than did satiated men. Although the effect size of this difference was small-to-moderate, it nevertheless suggests that there are significant differences in the attractiveness ratings based on breast size between hungry and satiated men. In addition, the results of this study corroborate previous work showing that hungry men rate a significantly heavier female body size as attractive [57]–[59]. Moreover, these results are in line with the findings of Study 1: in both studies, it appears to be the case that men who experience relative resource insecurity show a preference for a larger breast size than do men who experience resource security.

## General Discussion

It has been suggested that one function of female breast size is to act as an indicator of adipose tissue reserves in non-lactating women [15], [34]–[35]. This hypothesis is based on the fact that adipose tissue plays a central role in the storage of calories, which in turn leads to the suggestion that breast size may reliably predict food availability or access to resources. In situations marked by relative resource insecurity, then, men should idealise larger female breast size, as large size would indicate that a woman has access to resources. In two studies, we found evidence for this hypothesis: men who were experiencing relative resource insecurity (operationalised either as environmental socioeconomic context or proprioceptive hunger) rated women with larger breast sizes as more physically attractive than did men experiencing resource security.

Based on the present set of findings, it might be argued that temporary affective states produce individual variation in breast size judgements. Men experiencing immediate resource insecurity may perceive women with larger breasts as more attractive because large breast size indicates access to resources [57]–[59] or, more broadly, traits associated with maturity that may be more valued during periods of insecurity [60]–[65]. In short, the subjective experience of resource deprivation in the form of hunger appears to drive men to place greater value on female cues that indicate access to resources. Moreover, it is apparent that these temporary affective states mirror patterns of cross-environmental differences, with men from contexts of low socioeconomic status rating larger breast sizes as more attractive than men from contexts of high socioeconomic status. It is possible the cumulative temporal effect of resource insecurity among the former group is what drives their idealisation of a larger breast size [57], [59].

Of course, this is not to suggest that adipose tissue reserves are the only thing indicated by larger breast size. If this were the case, then larger breast size should be no more important than fat stored in any other part of a woman’s body [17]. Rather, breast size may also act as a cue of nulliparity, age, sexual maturity, or fertility [14]–[17] and, furthermore, there may be other more important cues of fat storage compared to the breasts, such as overall body size [57], [59]. This may help to explain the small-to-moderate effect sizes uncovered in both studies reported here: all things being equal breast size may indicate fat reserves, but in reality breast size is likely correlated with body mass [72], which may act as a more reliable indicator of such reserves. Determining the relative importance of breast size and body size, respectively, as cues of fat reserves will require further research.

Nor do our findings deny a role for sociocultural factors in shaping breast size judgements. It has been argued, for example, that breasts are one of the most important sites of objectification of the female body in socioeconomically developed settings [4], [72]–[73] and media targeted at some men appear to fetishise large breasts [74]–[75]. As an aside, this should not be used to suggest that the importance of breasts varies across cultures and that our methodology artificially inflates the importance of breast size: earlier ethnographic research indicates that breasts are eroticised in many different cultures [76]. In addition, judgements of breast size appear to be shaped by individual psychological differences [28], [30], as well as motivational states [77], which may help account for some of the discrepant findings in earlier studies. In future work, it will be important to take into account the different theoretical perspectives highlighted here in order to arrive at a fuller picture of the forces shaping breast size preferences across cultures.

There are a number of limitations of the present work, which should be recognised. First, it is possible that there were differences in mean breast size across our research sites (particularly in Study 1), which impacted on our respondents’ breast size preferences. For example, some scholars have suggested that attractiveness judgements are calibrated to local conditions [78]; this being the case, it is possible that local variations in mean breast size may have impacted upon men’s breast size judgements independent of socioeconomic status. Obtaining population-based anthropometric and tailoring stimuli according to local variation may help to expand on our findings. Second, it is possible that figures with larger breast size were perceived as heavier overall. If so, it is possible that our findings were driven by body size preferences in general, rather than breast size *per se*. Although variation in breast size in our stimuli is unlikely to have resulted in major in perceptions of body weight or size, this is an issue that warrants further investigation.

Third, our focus on breast size comes at the expense of other breast-related variables that may have impacted upon participants’ ratings, such as symmetry, shape, and areola size [7]. Although these traits were held constant in our study, future work may wish to concurrently consider the effects of manipulations to different breast-related variables, as well as other morphological traits, such as body size and waist-to-hip ratio. In a similar vein, because the faces of our stimuli were identical for each figure, participants may have focused more on the figures’ bodies as a result [7]. One way in which this limitation could be overcome would be to utilise a between-groups design in which participants are asked to rate only one figure, rather than being presented with all figures simultaneously.

These limitations notwithstanding, the present set of results provides evidence that breast size may play a role in men’s assessments of female access to resources. All things being equal, men from relatively low socioeconomic contexts and who experience temporary hunger rate women with larger breast size as more attractive than men from high socioeconomic contexts or are experiencing satiety. These results add to the findings of recent empirical work demonstrating the malleability of physical attractiveness ratings [65] and highlight the importance of considering the context in which attractiveness judgements are made. What remains is for scholars to begin the task of theorising how the many different factors that are known to impact upon physical attractiveness preferences (e.g., social, economic, evolutionary, individual differences) might fit together [79].

## References

[1] Swami V (2012) Physical attractiveness and personality. In Cash T, ed. Encyclopedia of body image and human appearance. Oxford, UK: Elsevier, 622–628.

[2] Swami V, Furnham A (2008) The psychology of physical attraction. London: Routledge.

[3] Swami V, Salem N (2011) The evolutionary psychology of human beauty. In Swami V, ed. Evolutionary psychology: A critical introduction. Oxford, UK: Wiley-Blackwell, 131–182.

[4] Dettwyler KA (1995) Beauty and the breast: The cultural context of breastfeeding in the United States. In MacAdam P, Dettwyler KA, eds. Breastfeeding: Biocultural perspectives. New York, NY: De Gruyter, 167–215.

[5] Koff E, Bevenage A (1998) Breast size perception and satisfaction, body image, and psychological functioning in Caucasian and Asian American college women. Sex Roles 38: 655–673.

[6] Cornelissen PL, Hancock PJB, Kiviniemi V, George HR, Tovée MJ (2009) Patterns of eye movements when male and female observers judge female attractiveness, body fat, and waist-to-hip ratio. Evol Hum Behav 30: 417–428.

[7] Dixson BJ, Grimshaw GM, Linklater WL, Dixson AF (2011) Eye-tracking of men’s preferences for waist-to-hip ratio and breast size of women. Arch Sex Behav 40: 43–50. 19688590 10.1007/s10508-009-9523-5

[8] Short RV (1980) The origins of human sexuality. In Austin CR, Short RV, eds. Reproduction in mammals, vol 8: Human sexuality. Cambridge, UK: Cambridge University Press, 1–33.

[9] Low BS, Alexander RD, Noonan KM (1987) Human hips, breasts, and buttocks: Is fat deceptive? Ethol Sociobiol 8: 249–257.

[10] Smith NW (1986) Psychology and evolution of breasts. J Hum Evol 1: 285–286.

[11] Einon D (2007) The shaping of women’s bodies: Men’s choice of fertility or heat stress avoidance? In Swami V, Furnham A, eds. The body beautiful: Evolutionary and sociocultural perspectives. Basingstoke, UK: Palgrave Macmillan, 131–158.

[12] Pawłowski B (1999) Permanent breasts as a side effect of subcutaneous fat increase in human evolution. Homo 50: 149–162.

[13] Morris D (1967) The naked ape: A zoologist’s study of the human animal. Toronto: Bantam Books.

[14] Barber N (1995) The evolutionary psychology of physical attractiveness: Sexual selection and human morphology. Ethol Sociobiol 16: 395–424.

[15] Gallup GG (1982) Permanent breast enlargement in human female: A sociobiological analysis. J Hum Evol 19: 111–123.

[16] Jasieńska G, Ziomkiewicz A, Ellison PT, Lipson SF, Thune I (2004) Large breasts and narrow waists indicate high reproductive potential in women. Proc Royal Soc B 271: 1213–1217. 10.1098/rspb.2004.2712 PMC1691716 15306344

[17] Marlowe F (1998) The nubility hypothesis: The human breast as an honest signal of residual reproductive value. Hum Nature 9: 263–271. 26197484 10.1007/s12110-998-1005-2

[18] Carter P (1996) Breast feeding and the social construction of heterosexuality, or ‘What breasts are really for’. In Holland J, Adkins L, eds. Sex, sensibility, and the gendered body. London: Macmillan, 99–119.

[19] Tantleff-Dunn S (2001) Breast and chest size: Ideals and stereotypes through the 1990s. Sex Roles 45: 231–242.

[20] Furnham A, Swami V (2007) Perceptions of female buttocks and breast size in profile. Soc Behav Pers 35: 1–8.

[21] Kleinke CL, Staneski R (1980) First impressions of female bust size. J Soc Psychol 110: 123–134.

[22] Horvath T (1981) Physical attractiveness: The influence of selected torso parameters. Arch Sex Behav 10: 21–24. 7212994 10.1007/BF01542671

[23] Tantleff-Dunn S (2002) Biggest isn’t always best: The effect of breast size on perceptions of women. J App Soc Psychol 32: 2253–2265.

[24] Wiggins JS, Wiggins N, Conger JC (1968) Correlates of heterosexual somatic preference. J Pers Soc Psychol 10: 82–90. 4386664 10.1037/h0026394

[25] Furnham A, Dias M, McClelland A (1998) The role of body weight, waist-to-hip ratio, and breast size in judgements of female attractiveness. Sex Roles 39: 311–326.

[26] Gitter AG, Lomranz J, Saxe L, Bar-Tal D (1983) Perception of female physique characteristics by American and Israeli students. J Soc Psychol 121: 7–13. 6645426 10.1080/00224545.1983.9924460

[27] Singh D, Young RK (1995) Body weight, waist-to-hip ratio, breasts, and hips: Role in judgements of attractiveness and desirability for relationships. Ethol Sociobiol 16: 483–507.

[28] Zelazniewicz AM, Pawłowski B (2010) Female breast size attractiveness for men as a function of sociosexual orientation (restricted versus unrestricted). Arch Sex Behav 40: 1129–1135. 10.1007/s10508-011-9850-1 PMC3210352 21975921

[29] Tovée MJ, Cornelissen PL (2001) Female and male perceptions of female physical attractiveness in front-view and profile. Br J Psychol 92: 391–402. 11417788

[30] Swami V, Tovée MJ (in press) Men’s oppressive beliefs predict their breast size preferences in women. Arch Sex Behav. 10.1007/s10508-013-0081-5 23412650

[31] Gray PB, Frederick DA (2012) Body image body type preferences in St Kitts, Caribbean: A cross-cultural comparison with US samples regarding attitudes towards muscularity, body fat, and breast size. Evol Psychol 10: 631–655. 22995446 10.1177/147470491201000319 PMC10426970

[32] Swami V, Jones J, Einon D, Furnham A (2009) Men’s preferences for women’s profile waist-to-hip ratio, breast size, and ethnic group in Britain and South Africa. Br J Psychol 100: 313–325. 18625082 10.1348/000712608X329525

[33] Dixson BJ, Vasey PL, Sagata K, Sibanda N, Linklater WL, et al (2010) Men’s preferences for women’s breast morphology in New Zealand, Samoa, and Papua New Guinea. Arch Sex Behav 40: 1271–1279. 20862533 10.1007/s10508-010-9680-6

[34] Cant J (1981) Hypothesis for the evolution of human breasts and buttocks. Am Naturalist 117: 199–204.

[35] Huss-Ashmore R (1980) Fat and fertility: Demographic implications of differential fat storage. Yearbook Physical Anthropol 23: 65–91. 10.1002/ajpa.1330230506 12264946

[36] Ramsay DT, Kent JC, Hartmann RA, Hartman PE (2005) Anatomy of the lactating human breast redefined with ultrasound imaging. J Anat 206: 525–534. 15960763 10.1111/j.1469-7580.2005.00417.x PMC1571528

[37] Bomalaski JJ, Tabano M, Hooper L, Fiorica J (2001) Mammography. Current Opinion Obstetrics Gyencol 13: 15–23. 10.1097/00001703-200102000-00003 11176228

[38] Tobon H, Salazar H (1975) Ultrastructure of the human mammary gland. II: Postpartum lactogenesis. J Clin Endocrinol Metab 40: 834–844. 1127090 10.1210/jcem-40-5-834

[39] Patton S, Jensen RG. (1976). Biomedical aspects of lactation. Oxford, UK: Pergamon Press.

[40] Hytten FE, Leitch I (1971) The physiology of human pregnancy, 2^nd^ ed. Oxford, UK: Blackwell.

[41] Niefert MRJ, Seacat JM, Jobe WE (1985) Lactation failure due to insufficient glandular development of the breast. Pediatrics 76: 823–828. 4058994

[42] Hsieh C, Trichopoulos D (1991) Breast size, handedness, and breast cancer risk. Euro J Cancer 27: 131–135. 10.1016/0277-5379(91)90469-t 1827274

[43] Trichopoulos D, Lipman E (1992) Mammary gland mass and breast cancer risk. Epidemiology 3: 523–526. 1420519 10.1097/00001648-199211000-00011

[44] Wade TD, Zhu G, Martin NG (2010) Body mass index and breast size in women: Same or different genes? Twin Res Hum Genet 13: 450–454. 20874466 10.1375/twin.13.5.450

[45] Swami V, Tovée MJ (2005) Female physical attractiveness in Britain and Malaysia: A cross-cultural study. Body Image 2: 115–128. 18089180 10.1016/j.bodyim.2005.02.002

[46] Tovée MJ, Swami V, Furnham A, Mangalparsad R (2006) Changing perceptions of attractiveness as observers are exposed to a different culture. Evol Hum Behav 27: 443–456.

[47] Swami V, Tovée MJ (2007) Differences in attractiveness preferences between observers in low and high resource environments in Thailand. J Evol Psychol 5: 149–160.

[48] Swami V, Tovée MJ (2007) Perceptions of female body weight and shape among indigenous and urban Europeans. Scand J Psychol 48: 43–50. 17257368 10.1111/j.1467-9450.2006.00526.x

[49] Swami V, Knight D, Tovée MJ, Davies P, Furnham A (2007) Perceptions of female body size in Britain and the South Pacific. Body Image 4: 219–223. 18089268 10.1016/j.bodyim.2007.01.002

[50] Swami V, Frederick DA, Aavik T, Alcalay L, Allik J, et al (2010) Body weight ideals and body dissatisfaction in 26 countries across 10 world regions: Results of the International Body Project I. Pers Soc Psychol Bull. 36: 309–325. 10.1177/0146167209359702 20179313

[51] Swami V, Tovée MJ (2005) Male physical attractiveness in Britain and Malaysia: A cross-cultural study. Body Image 2: 383–393. 18089203 10.1016/j.bodyim.2005.08.001

[52] Government of Malaysia (2001) The Eight Malaysia Plan, 2001–2005. Putrajaya, Malaysia: Economic Planning Unit, Prime Minister’s Department.

[53] Swami V, Tovée MJ (2007) The relative contribution of profile body shape and weight to judgements of women’s physical attractiveness in Britain and Malaysia. Body Image 4: 391–396. 18089286 10.1016/j.bodyim.2007.07.002

[54] Swami V, Kannan K, Furnham A (2012) Positive body image: Inter-ethnic and rural-urban differences among an indigenous sample from Malaysian Borneo. Int J Soc Psychol 58: 568–576. 10.1177/0020764011415208 21821633

[55] Swami V, Furnham A (2010) Self-assessed intelligence: Inter-ethnic, rural-urban, and sex differences in Malaysia. Learn Individ Differ 20: 51–55.

[56] Halik M, Webley P (2011) Adolescents’ understanding of poverty and the poor in rural Malaysia. J Econ Psychol 32: 231–239.

[57] Nelson LD, Morrison EL (2005) The symptoms of resource scarcity: Judgements of food and finances influence preference for potential partners. Psychol Sci 16: 167–173. 15686584 10.1111/j.0956-7976.2005.00798.x

[58] Pettijohn TFII, Sacco DF Jr, Yerkes MJ (2009) Hungry people prefer more mature mates: A field test of the environmental security hypothesis. J Soc Evol Cultural Psychol 3: 216–232.

[59] Swami V, Tovée MJ (2006) Does hunger influence judgements of female physical attractiveness? Br J Psychol 97: 353–363. 16848948 10.1348/000712605X80713

[60] Pettijohn TFII, Tesser A (1999) An investigation of popularity in environmental context: Facial feature assessment of American movie actresses. Media Psychol 1: 229–247.

[61] Pettijohn TFII, Tesser A (2003) History and facial features: The eyes have it for actresses but not for actors. North Am J Psychol 5: 335–345.

[62] Nelson LD, Pettijohn TF II, Galak J (2007) Mate preferences in social cognitive context: When environmental and personal change leads to predictable cross-cultural variation. In: Swami V, Furnham A, eds. Body beautiful: Evolutionary and socio-cultural perspectives. Basingstoke: Palgrave Macmillan, 185–206.

[63] Pettijohn TFII, Jungeberg B (2004) *Playboy* playmate curves: Changes in facial and body feature preferences across US social and economic conditions. Pers Soc Psychol Bull 30: 1186–1997. 15359021 10.1177/0146167204264078

[64] Pettijohn TFII, Tesser A (2005) Threat and social choice: When eye size matters. J Soc Psychol 145: 547–570. 16201678 10.3200/SOCP.145.5.547-570

[65] Swami V, Tovée MH (2012) The impact of psychological stress on men’s judgements of female body size. PLoS ONE 7: e42593. 22905153 10.1371/journal.pone.0042593 PMC3414440

[66] Ortigosa A, Rowe L (2002) The effect of hunger on mating behavior and sexual selection for male body size in *Gerris buenoi* . Animal Behav 64: 369–375.

[67] Swami V, Poulogianni K, Furnham A (2006) The influence of resource availability on preferences for human body weight and non-human objects. J Articles Supp Null Hyp 4: 17–28.

[68] Flint A, Raben A, Blundell JE, Astrup A (2000) Reproducibility, power, and validity of visual analogue scales in assessment of appetite sensations in single test meal studies. Int J Obesity 24: 38–48. 10.1038/sj.ijo.0801083 10702749

[69] Merrill EP, Kramer FM, Cardello A, Schultz H (2002) A comparison of satiety measures. Appetite 39: 181–183. 12354688 10.1006/appe.2002.0496

[70] Niedhammer I, Bugel I, Bonenfant S, Goldberg M, Leclerc A (2000) Validity of self-reported height and weight in the French GAZEL cohort. Int J Obes Relat Metab Disord 24: 1111–1118. 11033979 10.1038/sj.ijo.0801375

[71] Spencer EA, Appleby PN, Davey GK, Key TJ (2002) Validity of self-reported height and weight in 4808 EPIC-Oxford participants. Public Health Nutr 5: 561–565. 12186665 10.1079/PHN2001322

[72] Brown N, White J, Milligan A, Risius D, Ayres B, et al (2012) The relationship between breast size and anthropometric characteristics. Am J Hum Biol 24: 158–164. 22287066 10.1002/ajhb.22212

[73] Seifert T (2005) Anthropometric characteristics of centerfold models: Trends towards slender figures over time. Int J Eat Disord 37: 271–274. 15822083 10.1002/eat.20086

[74] Ward LM, Merriwether A, Caruthers A (2006) Breasts are for men: Media, masculinity ideologies, and men’s beliefs about women’s bodies. Sex Roles 55: 703–714.

[75] Gerald W, Potvin L (2009) Boobs, boxing, and bombs: Problematising the entertainment of Spike TV. Spaces Diff 2: 3–14.

[76] den Daas C, Häfner M, de Wit J (in press) Sizing opportunity: Biases in estimates of goal-relevant objects depend on goal congruence. Soc Psychological Pers Sci.

[77] Ford CS, Beach FA (1952) Patterns of sexual behavior. New York, NY: Harper.

[78] Sugiyama LS (2004) Is beauty in the context-sensitive adaptations of the beholder? Shiwiar use of waist-to-hip ratio in assessments of female mate value. Evol Human Behav 25: 51–62.

[79] Swami V, Furnham A, ed. (2007). Body beautiful: Evolutionary and socio-cultural perspectives. Basingstoke: Palgrave Macmillan.

